# Reply to: Target expression is a relevant factor in synthetic lethal screens

**DOI:** 10.1038/s42003-022-03747-5

**Published:** 2022-08-19

**Authors:** Yosi Gilad, Yossi Eliaz, Yang Yu, Adam M. Dean, San Jung Han, Li Qin, Bert W. O’Malley, David M. Lonard

**Affiliations:** 1 Department of Molecular and Cellular Biology, Baylor College of Medicine, Houston, TX USA; 2 Department of Molecular and Human Genetics, Baylor College of Medicine, Houston, TX USA

**Keywords:** Drug screening, Cancer screening, Breast cancer

**replying to** I.J. Schultz & H.J.T. Coelingh Bennink *Commun. Biol.* 10.1038/s42003-022-03746-6 (2022)

The major concern that the authors have risen is the enhanced sensitivity of BC cell lines to SI-12 treatment in our study as a result of the silencing of four genes—*OR4D6*, *OR5I1*, *NDNF*, and *S1PR1*—in spite of the lack of their expression in these cell lines, according to public databases and previously published studies.

There are a few points that might explain this contradiction:

- It is important to mention that in the studies by Weber et al. ^1^ and Masjedi et al. ^2^, that the authors have mentioned, the transcriptome analysis was performed on untreated samples, while the importance of *OR5I1* and *OR4D6* in the context of our study manifested under SI-12 treatment.
- We have performed qPCR experiments to access the mRNA levels following siRNA treatment in MCF7 cells. We would like to mention that the apparent lack of concordance between our RT-qPCR data and DepMap RNA-Seq data could be due to false-negative discovery, which is a common issue of RNA-Seq^3,4^, and that RT-qPCR is capable of greater sensitivity^5^. Moreover, it is well known that many key driver genes, including signaling receptors, are frequently expressed at very low levels in cancer cells^6,7^ and therefore are even more prone for false-negative discovery by RNA-Seq.
- There are evidences that the expression of OR varies across individuals^8^; therefore it might be also the case across cell line subsets.
- It is an unfortunate, but inevitable nature of cancer cell lines that have the same parental origin, but which had been handled differently (in the case of MCF7 cells - for decades), to have a non-homogenous gene-expression profile. In the context of the discussed four genes, we found that it might be best exemplified by the fact that while the expression of *S1PR1* is absent in MCF7 cells, as you have found by interrogation of publically available databases, this gene was found to be expressed in MCF7 cells elsewhere. Furthermore, it was found to have functional importance in this cell line^9–12^.

Importantly, since the main goal our study was to find gene targets that can be pharmacologically inhibited, the focus of our study was made on screening candidates that could be validated not only by siRNA perturbation, but also by pharmacological inhibition. However, the authors of this MA focused on four genes, three of which could not be pharmacologically validated (*OR4D6*, *OR5I1*, and *NDNF*, since, to the best of our knowledge, there are no available pharmacological inhibitors for these three genes). Therefore, we made a clear statement in our paper that these genes are potential anti-cancer therapeutic targets in combination with SI-12 rather than validated ones. The only target out of the four genes that are in the focus of the discussion of this MA that has been validated both by an individual siRNA perturbation and pharmacological inhibition in combination with SI-12 is S1PR1. Indeed, our results show that the pharmacological inhibition of S1PR1 did not contribute to the cancer cell-killing effect in combination with SI-12 (which might support the author's concern regarding a robust and biologically significant expression of this gene). Therefore, this target was not included in our conclusive remarks as a valid target for combination treatment with SI-12. We would like to mention that the lack of complete agreement between initial screening candidates and later individual validations is almost a matter of fact in these types of experiments, and generally, this is why validation experiments are carried out following screening.
